# Basal cortisol in relation to metyrapone confirmation in predicting adrenal insufficiency after pituitary surgery

**DOI:** 10.1007/s11102-023-01374-9

**Published:** 2024-01-19

**Authors:** Pieter E. Huisman, Sarah E. Siegelaar, Jantien Hoogmoed, René Post, Shariefa Peters, Moniek Houben, Jacquelien J. Hillebrand, Peter H. Bisschop, Alberto M. Pereira, Eveline Bruinstroop

**Affiliations:** 1 Department of Endocrinology and Metabolism, Amsterdam UMC, Location University of Amsterdam, Amsterdam, The Netherlands; 2 Department of Neurosurgery, Amsterdam UMC Location University of Amsterdam, Meibergdreef 9, Amsterdam, The Netherlands; 3 Endocrine Laboratory, Department of Laboratory Medicine, Amsterdam UMC, Location University of Amsterdam, Meibergdreef 9, Amsterdam, The Netherlands; 4 Pituitary Center Amsterdam, Amsterdam, The Netherlands; 5 Amsterdam Gastroenterology Endocrinology Metabolism, Amsterdam, The Netherlands; 6 Department of Neurosurgery, Neurosurgical Center Amsterdam, Amsterdam, The Netherlands; 7 Cancer Center Amsterdam, Amsterdam, The Netherlands

**Keywords:** Transsphenoidal surgery, Cortisol, Hydrocortisone replacement, Metyrapon, Adrenal insufficiency

## Abstract

**Purpose:**

Pituitary surgery can lead to post-surgical adrenal insufficiency with the need for glucocorticoid replacement and significant disease related burden. In patients who do not receive hydrocortisone replacement before surgery, at our center, an early morning plasma cortisol concentration using a cut-off value of 450 nmol/L 3 days after surgery (POD3) is used to guide the need for hydrocortisone replacement until dynamic confirmatory testing using metyrapone. The aim of this study was to critically assess the currently used diagnostic and treatment algorithm in patients undergoing pituitary surgery in our pituitary reference center.

**Methods:**

Retrospective analysis of all patients with a POD3 plasma cortisol concentration < 450 nmol/L who received hydrocortisone replacement and a metyrapone test after 3 months. Plasma cortisol concentration was measured using an electrochemiluminescence immunoassay (Roche). All patients who underwent postoperative testing using metyrapone at Amsterdam UMC between January 2018 and February 2022 were included. Patients with Cushing’s disease or those with hydrocortisone replacement prior to surgery were excluded.

**Results:**

Ninety-five patients were included in the analysis. The postoperative cortisol concentration above which no patient had adrenal insufficiency (i.e. 11-deoxycortisol > 200 nmol/L) was 357 nmol/L (Sensitivity 100%, Specificity 31%, PPV:32%, NPV:100%). This translates into a 28% reduction in the need for hydrocortisone replacement compared with the presently used cortisol cut-off value of 450 nmol/L.

**Conclusion:**

Early morning plasma cortisol cut-off values lower than 450 nmol/L can safely be used to guide the need for hydrocortisone replacement after pituitary surgery.

## Introduction

Pituitary surgery is the preferred treatment for pituitary masses in case of mass effect of the tumor on the opticochiasmatic apparatus or in most of the functional adenomas. Post-surgical adrenal insufficiency may occur and is potentially life-threatening. Consequently, the assessment of the activity of the hypothalamic pituitary adrenal axis (HPA-axis) after surgery is very important. There are no guidelines and there is no consensus on how and when to test for adrenal insufficiency after surgery, resulting in the use of different strategies based on institutional protocols. In patient with normal adrenal function prior to surgery, most protocols include measuring an early morning cortisol after pituitary surgery, in case of perioperative glucocorticoid replacement, after withdrawal of hydrocortisone for at least 12 h. The use of perioperative glucocorticoid replacement is still a matter of debate [1]. There are centers that do not administer any glucocorticoids perioperatively [2]. At our pituitary center, patients receive per protocol multiple doses of hydrocortisone in the perioperative setting to prevent secondary adrenal insufficiency.

An early morning cortisol of > 450 nmol/L indicates normal activity of the HPA-axis [3], whereas cortisol concentrations below 450 nmol/L may reflect the presence of adrenal insufficiency with the need for hydrocortisone replacement. These patients require further dynamic testing to confirm or reject the presence of adrenal insufficiency. The optimal early morning postoperative cortisol cut-off value is currently still a matter of debate. A lower cut-off value may reduce unnecessary hydrocortisone replacement and related burden at the expense of possibly missing a diagnosis of adrenal insufficiency [4].

A limited number of single center studies have proposed lower cut-off values than 450 nmol/L with cortisol concentrations exceeding 350 nmol/L being already highly suggestive of normal adrenal reserve [3, 5, 6]. Most studies have used the insuline tolerance test (ITT) 4 weeks to even months after surgery as the gold standard to confirm or reject a definite diagnosis of adrenal insufficiency. However, to date, no studies have reported on the predictive value of postoperative cortisol concentrations using the metyrapone test. At our tertiary pituitary referral center, a metyrapone test is performed approximately 3 months after surgery to confirm or reject a diagnosis of adrenal insufficiency. This may also be of added value as previous studies suggested that the measurement of adrenocorticotropic hormone (ACTH) adds sensitivity (subjects with a subnormal ACTH response, but adequate 11-deoxycortisol response, being labeled as having partial ACTH deficiency) [7].

The metyrapone test has been established as a safe and reliable test to determine adrenal insufficiency [8]. In our pituitary center there is long-term experience with this test which was internally validated within our center [9]. Although there are side effects of vomiting and nausea and require the patients to stay overnight we use this test post-operatively as a reliable and patient friendly test opposed to the insulin tolerance test (ITT) with known risks during the test and contra-indications such as coronary artery disease and epilepsy. Also, the ITT has large variability in the required dose in insulin resistant subjects and causes significant discomfort in most patients [10].

In this study we aimed to retrospectively assess the diagnostic accuracy of our protocol and investigated possible interfering factors (e.g. use of perioperative dexamethasone) in addition to clinical measurement to strengthen the predictive value. Application of an optimal cortisol cut-off value will prevent unnecessary hydrocortisone replacement with avoidable healthcare costs and, more importantly, improve quality of live, by reducing medication use and its accompanying requirements regarding stress instructions on how and when to adjust and use oral and parenteral hydrocortisone [4, 11].

## Methods

### Data collection

Ethical approval was waived by the Medical Ethics Review Committee of the Academic Medical Center in view of the retrospective nature of the study. A retrospective chart review was performed of all patients who underwent postoperative metyrapone testing at our center. Patients who underwent transsphenoidal pituitary or transcranial surgery between January 2018 and February 2022 were included who received a postoperative measurement of cortisol and a subsequent metyrapone test approximately 3 months after surgery. This time period was chosen as a different cortisol assay was used before 2018. Exclusion criteria were preoperative use of hydrocortisone and patients with Cushing’s disease. At our center, patients with postoperative cortisol values > 450 nmol/L do not undergo a metyrapone test, and were therefore not included in this study, except for three cases in which a metyrapone test was performed because of other clinical indications.

Data were extracted from the electronic patient records. Endocrine analyses have in most cases been performed in the referring center and therefore patients are referred with or without hydrocortisone replacement based on basal cortisol combined with complaints and/or dynamic testing. During the first visit in our center and for every new patient diagnosed in our center we evaluate the endocrine analysis and perform, if necessary, additional preoperative assessment of pituitary hormone axes. Preoperative pituitary insufficiency was noted when defined as insufficiency by the treating physician and/or receiving treatment for deficiency. Postoperative diabetes insipidus was defined as need for at least one dose of desmopressin during hospitalization. Tumor size was noted only as < 10 mm or ≥ 10 mm. The data were extracted by the first author [12] and discussed with the last author when needed. Thereafter, a data check was performed by the last author [13].

### Perioperative protocol

Patients not receiving hydrocortisone replacement before surgery started with hydrocortisone on the day of surgery (day 0) with 50 mg one hour before surgery and two subsequent dosages of 50 mg that same day. On the first postoperative day (POD1) patients received 25 mg hydrocortisone (iv injection) three times a day. On POD2 patients only received a single oral dose of 20 mg of hydrocortisone in the morning with no subsequent dosages. On POD3 an early morning cortisol concentration was measured between 6 and 8 AM, prior to the morning intake of hydrocortisone. Blood was collected by vena punction. Patients with a basal cortisol < 450 nmol/L continued hydrocortisone replacement, and received stress instructions in the hospital to prevent an adrenal crisis prior to discharge (including hydrocortisone for parenteral use in case of emergency) and an appointment after discharge at the specialized endocrine unit with the endocrine specialist nurses for further explanations regarding practical do’s and don’ts in case of stress. The replacement dose given to patients with low cortisol concentrations (< 450 nmol/L) on the third postoperative day was 20 mg per day, divided into three doses during the day (10 mg at 8 AM, 5 mg at 12 AM and another 5 mg at 5 PM). Patients with cortisol concentrations > 450 nmol/L were discharged without hydrocortisone and no follow-up test was routinely performed. Some patients also received a dose of dexamethasone (4 or 8 mg) during anesthesia to prevent postoperative nausea.

### Metyrapone test

Per protocol, a metyrapone test was performed 3 months after surgery in all patients with POD3 cortisol < 450 nmol/L. When applicable, patients were instructed to discontinue estrogen containing medication (oral contraceptives) 6 weeks before the test. Hydrocortisone was discontinued 24 h prior to the test. Patients were admitted to the hospital and were not allowed to eat or drink after 10 PM. Metyrapone was given orally at 11:30 PM [2.0 g (body weight < 70 kg), 2.5 g (70–90 kg), or 3.0 g (> 90 kg)]. The next morning (8–9 AM), blood was collected by vena punction. 11-deoxycortisol (serum), ACTH (EDTA plasma) and cortisol (heparin plasma) were determined. Patients were diagnosed with adrenal insufficiency, when 11-deoxycortisol concentration after metyrapone was ≤ 200 nmol/L.

### Assay type and specifications

The immunoassay for serum cortisol was performed on a Roche cobas e602 immunochemistry analyzer (Elecsys Cortisol II, Roche, Mannheim, Germany). The inter-assay coefficients of variation for means 176 and 901 nmol/L were 2.8% and 2.0% respectively. The limit of quantitation was 3.0 nmol/L, but concentrations below 30 nmol/L were not specified and reported as < 30 nmol/L.

11-deoxycortisol values were determined on a LC–MS/MS (Waters, Milford MA, U.S.A.). The inter-assay coefficient of variation (CV) was 2.8% (mean 1.0 nmol/L) and 0.9% (mean 286 nmol/L). The limit of quantitation was 0.5 nmol/L. Values above 400 nmol/L were not further specified.

ACTH values were measured on a Liaison XL chemiluminescence analyzer by DiaSorin (DiaSorin, Saluggia, Italy). The inter-assay CVs were 4.9% and 4.4% for their respective means 9.5 pmol/L and 65 pmol/L. The limit of quantitation was 0.84 pmol/L.

### Statistical analysis

Statistical analysis was performed with IBM SPSS Statistics 28 (IBM Corp. Armonk, NY, USA). The cortisol concentration above which all metyrapone tests were normal (sensitivity = 1; 11-deoxycortisol > 200 nmol/L) and below which all metyrapone tests were abnormal (specificity = 1; 11-deoxycortisol ≤ 200 nmol/L) was determined. Both sensitivity and specificity, as well as the positive predictive value (PPV) and negative predictive value (NPV) were calculated for different cut-off values.

Furthermore, binary logistic regression was performed to determine whether the timing of the metyrapone test was associated with the outcome of metyrapone test. After performing the Shapiro–Wilk test to check for normality, the mean cortisol value between patients who did and did not receive dexamethasone during operation were compared using the independent samples t test.

Finally, binary logistic regression was performed to assess if certain patient characteristics (e.g., sex, tumor type or tumor size) are associated with a higher risk of developing adrenal insufficiency. A p value below 0.05 was considered statistically significant.

## Results

### Patient selection

**Fig. 1:**
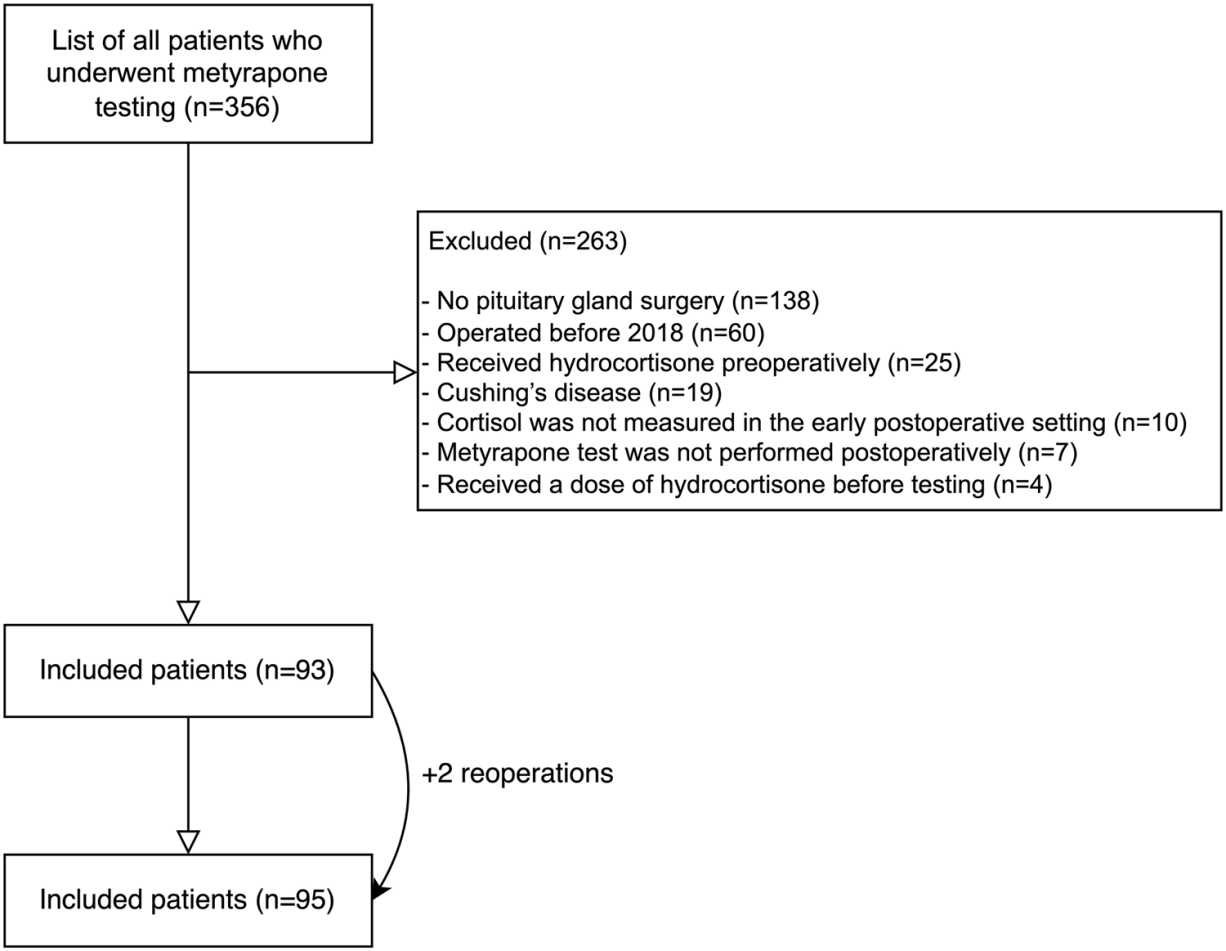
Flowchart patient selection

A total of 356 patient charts were reviewed (Fig. 1). Based on the previously mentioned selection criteria, a total of 93 patients were included. Two patients were included twice because they underwent two surgeries. No patients received radiotherapy before surgery.

The following patients were excluded: patients that did not undergo pituitary gland surgery (n = 138), patients that were operated before 2018 [use of different cortisol assay (n = 60)], patients that received hydrocortisone preoperatively (n = 25), patients with Cushing’s disease (n = 19), patients in whom cortisol was not measured in the early postoperative setting (n = 10), or the metyrapone test was not performed postoperatively (n = 7). In addition, there were some cases (n = 8) in which patient’s serum cortisol was not determined on POD3, but on POD2-POD7. In these cases, it was checked whether patients indeed received hydrocortisone only in the morning on the prior day resulting in the exclusion of four patients for which this protocol was not followed.

### Patient characteristics

A summary of patient characteristics can be found in Table 1. The male/female ratio was 43/52 and the mean age 53 years (SD 14). In this patient cohort with basal cortisol after surgery of < 450 nmol/L, 24% of the cases (n = 23) was diagnosed with adrenal insufficiency by metyrapone testing. The median time between surgery and the metyrapone test was 14 weeks (range 52; IQR 4). In 32% of cases (n = 30) dexamethasone was administered during surgery.

**Table 1:** Patient characteristics

Characteristics	n (%)
Total patients	95
Male	43 (45)
Female	52 (55)
Mean age (years)	53 (SD 14)
Type of surgery	
Transsphenoidal	89 (94)
Transcranial	6 (6)
Reoperation	
Yes	13 (14)
No	82 (86)
Tumor size	
Microadenoma (< 10 mm)	9 (9)
Macroadenoma (≥ 10 mm)	86 (91)
Tumor type	
Non-functioning adenoma	53 (56)
GH-secreting adenoma	19 (20)
Prolactinoma	5 (5)
TSH-secreting adenoma	2 (2)
Craniopharyngioma	6 (6)
Meningioma	5 (5)
Cysts	5 (5)
Preoperative pituitary hormone deficiency	
No deficiencies	69 (73)
A single deficiency	19 (20)
Multiple deficiencies	7 (7)
Cortisol measured on POD_x_	
POD_2_	1 (1)
POD_3_	91 (96)
POD_4_	2 (2)
POD_7_	1 (1)
Median timing metyrapone test (weeks)	14 (range 5–57)
Adrenal insufficiency (metyrapone test)	23 (24)
Dexamethasone administration during operation	30 (31)
Diabetes insipidus during hospitalization	23 (24)

Values are depicted as n (%) unless otherwise specified

### Diagnostic accuracy of POD3 cortisol

**Fig. 2:**
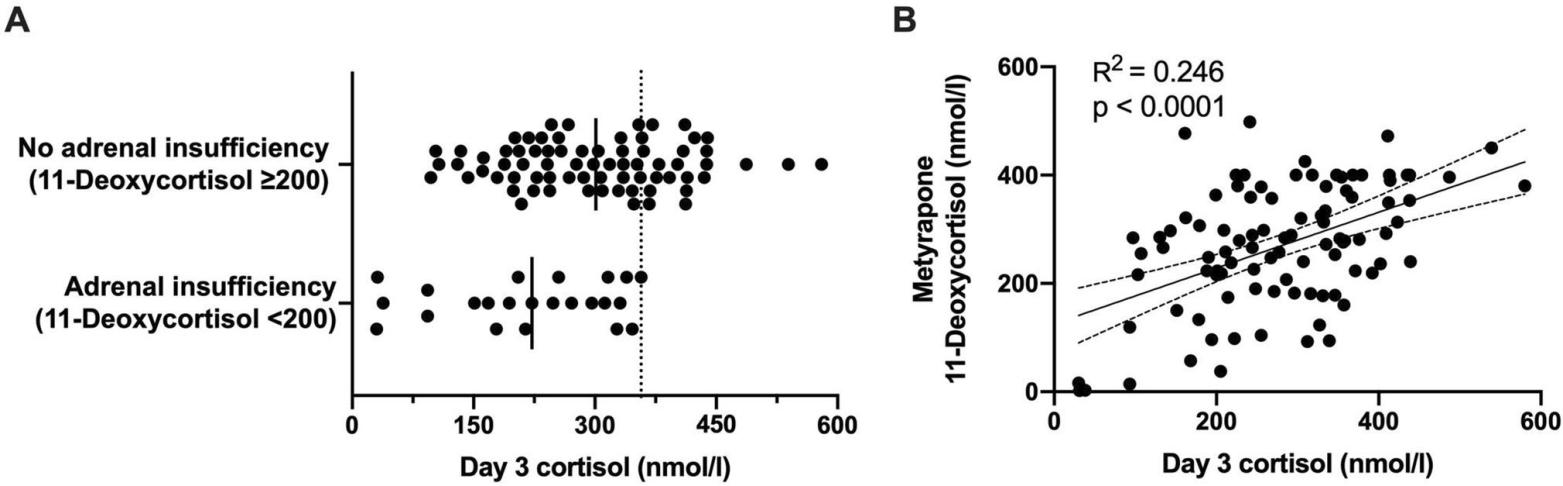
**A** Cortisol values sorted by adrenal function (dotted line = 357 nmol/l). **B** Correlation between 11-deoxycortisol and POD3 cortisol concentration

No patients with a basal postoperative cortisol > 357 nmol/L (n = 22) were diagnosed with adrenal insufficiency using the metyrapone test (Fig. 2). All patients with a basal postoperative cortisol < 97 nmol (n = 5) were diagnosed with adrenal insufficiency. The positive predictive value (PPV) of postoperative cortisol using a cut-off of 450 nmol/L was 25%. Seventy-five percent of patients therefore discontinued hydrocortisone replacement after 3 months. For every single patient continuing treatment, 3.0 patients could discontinue treatment (NNT = 3.0). A cut-off value of 357 nmol/L corresponded with a sensitivity and specificity of 100% and 31%, respectively (PPV 32%; NPV 100%). With this cut-off value, the NNT would be 2.2. An overview of the diagnostic performance for different cut-off values is given in Table 2.

**Table 2:** Summary of diagnostic performance for different cut-off values

Cut-off value (nmol/L)	Sensitivity	Specificity	PPV	NPV	% of pts receiving unnecessary medication^a^	% of pts being spared unnecessary treatment^a^	% of missed diagnoses^b^
357	1	0.31	0.32	1	72 (50/69)	28 (19/69)	0
300	0.70	0.50	0.31	0.84	52 (36/69)	48 (33/69)	30 (7/23)
250	0.57	0.63	0.33	0.82	39 (27/69)	61 (42/69)	43 (10/23)
200	0.39	0.82	0.41	0.81	19 (13/69)	81 (56/69)	61 (14/23)

^a^Compared to the amount of patients who received unnecessary treatment at a cut-off value of 450 nmol/L

^b^Compared to all diagnoses of adrenal insufficiency

A significant association between postoperative cortisol concentrations and 11-deoxycortisol (R^2^ = 0.246; p > 0.0001) and ACTH (R^2^ = 0.069; p = 0.01) during metyrapone was found (Fig. 2). 11-deoxycortisol concentrations and ACTH during the metyrapone test were significantly correlated (R^2^ = 0.201; p > 0.0001). Regression analysis showed that the timing of the metyrapone test did not interfere with the outcome of the metyrapone test (p = 0.71).

### Influence of dexamethasone administration on POD3 cortisol

In this cohort, 30/95 patients received a single dose of dexamethasone during surgery at a dose of 4 mg (n = 27) or 8 mg (n = 3). Blood samples were taken at least 62 h (median 68 h; IQR 4) after dexamethasone administration. Mean basal cortisol values in this group treated with dexamethasone were not statistically different from patients not receiving dexamethasone treatment (dexamethasone: 287 ± 132; no dexamethasone 274 ± 99; Mean ± SD; p = 0.60). Seventy-four percent of patients who received dexamethasone were falsely started with hydrocortisone supplementation, (i.e. they had a POD3 cortisol concentration < 450 nmol/L and 11-deoxycortisol > 200), versus 75% of patients who did not receive dexamethasone. We did not observe differences in PPVs between patients who did and those who did not receive dexamethasone.

### Multivariate regression analysis of clinical factors

In multivariate regression analysis we used a step-wise approach investigating whether additional independent variables were able to increase the accuracy of basal cortisol to predict adrenal insufficiency diagnosed by metyrapone testing. Both the outcome of the test, adrenal insufficiency defined as 11-deoxycortisol ≤ 200 nmol/L, and the continuous value of 11-deoxycortisol were used for this analysis (Table 3). Only postoperative basal cortisol showed a significant association with the outcome adrenal insufficiency (p = 0.003) and 11-deoxycortisol (p < 0.001). Reoperation showed a baseline significance with a trend toward increased incidence of adrenal insufficiency (p = 0.055). No multivariate model could be built to strengthen the predictive value of basal cortisol.

**Table 3:** Individual parameters and their corresponding p values after regression analysis

Parameter	Nagelkerke R^2^ (adrenal insufficiency)	Beta coefficient (11-deoxycortisol)	p value (adrenal insufficiency)	p value (11-deoxycortisol)
Age	0.000	− 0.088	0.975	0.394
Sex	0.007	0.009	0.498	0.934
Postoperative cortisol value (nmol/L)	0.149	0.496	0.003	< 0.001
Time between surgery and metyrapone test (weeks)	0.002	− 0.015	0.708	0.883
Dexamethasone administration (n = 30)	0.000	− 0.041	0.861	0.696
Type of operation (transsphenoidal/transcranial)	0.004	0.041	0.593	0.696
Reoperation (n = 13)	0.054	0.079	0.055	0.444
Tumor type				
Non-functioning adenoma^a^ (n = 53)	0.005	0.073	0.574	0.479
GH-secreting adenoma^a^ (n = 19)	0.043	− 0.074	0.136	0.474
Prolactinoma^a^ (n = 5)	0.044	− 0.149	0.999	0.149
TSH-secreting adenoma (n = 2)	0.010	− 0.124	0.414	0.230
Craniopharyngioma^a^ (n = 6)	0.031	0.112	0.148	0.281
Meningioma^a^ (n = 5)	0.010	− 0.022	0.407	0.835
Cysts^a^ (n = 5)	0.001	− 0.104	0.822	0.314
Tumor size (< or ≥ 10 mm)	0.017	− 0.042	0.353	0.690
Preoperative pituitary deficiency^a^				
No pituitary deficiency (n = 69)	0.031	− 0.156	0.151	0.130
Single pituitary deficiency (n = 19)	0.030	0.133	0.157	0.199
Multiple pituitary deficiencies (n = 7)	0.001	0.063	0.780	0.542
Diabetes insipidus (n = 23)	0.010	0.098	0.425	0.696

^a^Each individual category is compared to the other categories combined

## Discussion

In this study, we demonstrate that a cortisol concentration of 357 nmol/L measured 3 days after surgery can safely be used to guide post-surgical hydrocortisone replacement in patients with pituitary tumors until confirmatory testing of adrenal function with metyrapone is performed. The cut-off value of 450 nmol/L results in overtreatment of a significant number of patients in our patient cohort. In our study, a cut-off value of 357 nmol/L corresponds to a sensitivity of 100%. Using this new cut-off value, 28% of patients (n = 19) would have been spared unnecessary treatment. Of note, patients with high postoperative cortisol values after surgery (> 450 nmol/L) were not subjected to metyrapone testing, and were therefore not included in this study. Consequently, the incidence of adrenal insufficiency in this study is not representative for the entire cohort of patients who underwent pituitary surgery at our center during the study period.

A limited number of previous studies have proposed even lower cut-off values using the ITT as golden standard. De Vries et al. reported a cortisol cut-off value of 325 nmol/L with 100% sensitivity [6] (compared to ITT after a mean of 8 months), measured on an electrochemiluminescent immunoassay (Roche cobas 8000 module e602). English et al. reported an optimal cut-off value of 320 nmol/L (determined on a Beckman DxI800 immunoassay analyser) with a sensitivity of 84% (CI 60–97) (cortisol measured on POD3 or POD4, compared to glucocorticoid requirement after 6 months) [8]. Application of a cut-off of 325 nmol/L in our study would result in 5 patients with adrenal insufficiency that would have been missed (leading to a sensitivity of 78%). Comparisons with other studies, however, are difficult, as many factors can differ: the type of assay used, the use of different confirmation tests, differences in the number of days after surgery on which cortisol concentrations are determined, the heterogeneity of the patient cohort, and timing of the confirmation test. However, previous reports clearly indicate that the metyrapone test identifies more patients with subtle ACTH deficiency with unknown clinical significance [14]. This could, at least in part, explain the higher cut-off value of POD3 cortisol found in our study. Concerning the metyrapone test, an 11-deoxycortisol concentration of more than 200 nmol/L (7 μg/dL) is considered a normal response [9]. In terms of ACTH response, postoperative morning cortisol values correlated significantly with ACTH values, however this correlation was not as significant as 11-deoxycortisol concentrations (data not shown).

The use of dexamethasone during surgery may potentially affect post-surgical cortisol levels. In our tertiary referral center, blood samples were taken at least 62 h after dexamethasone administration during surgery and we found no differences in cortisol concentrations between the patient that received, and those that did not receive dexamethasone. In accordance with our findings, a previous study reported that maximal cortisol suppression was observed 24 h after the intravenous administration of 8 mg dexamethasone [15], and after 47 h, the cortisol values of the placebo group did not differ significantly from the control group. However, when the cortisol concentrations are measured earlier (than POD3) this could possibly affect the results, which should be taken into consideration when evaluating lab results. Therefore, each reference center should apply institutionally based cut-off values based on own test results [6].

In all of our patients, hydrocortisone was administered perioperatively (50 mg one hour before surgery and two subsequent dosages of 50 mg that same day, 75 mg intravenously on POD1, 20 mg orally on POD2) including those without preoperative adrenal insufficiency. Perioperative glucocorticoid supplementation has been the standard of care for a long period of time [16]. There is, however, debate whether this protocol is still necessary [1]. Some studies have challenged the use of perioperative glucocorticoid replacement as the incidence of secondary adrenal insufficiency and other postoperative complications did not differ significantly between patients with and without perioperative intravenous steroid administration, as is shown in a retrospective study with nearly two thousand cases [1, 17]. Some centers already implement a glucocorticoid sparing protocol with close monitoring for adrenal insufficiency [2]. A recent, triple-masked, randomized clinical trial showed that the incidence of new-onset adrenal insufficiency directly after surgery was 11% (95% CI 6.9–15.2%) in the non-hydrocortisone group compared to 6.4% (95% CI 3.2–9.7%) in the hydrocortisone group which did not reach statistical significance. In a subgroup analysis of patients with a BMI < 25.5 kg/m^2^ there was a significant difference in post-operative adrenal insufficiency [16]. In this study the reduction of hydrocortisone related complication of diabetes together with incidence of new-onset hypokalemia, hypernatremia and hypocalcemia was reduced by withholding hydrocortisone whereas incidence of hyponatremia was significantly increased by withholding treatment. Based on these studies the perioperative administration of hydrocortisone to patients with normal adrenal function preoperatively may be too conservative when serial morning cortisol measurements and strict monitoring for adrenal insufficiency related symptoms are in place which can be managed adequately and will not cause additional morbidity.

It has also been debated whether a 100% sensitivity should be the ultimate goal or whether a lower cut-off with close monitoring for symptoms of adrenal insufficiency can also be used. Such a strategy would further reduce hydrocortisone prescriptions. Inder et al. proposed an algorithm in which only patients with a cortisol < 250 nmol/L would receive daily hydrocortisone replacement and patients with cortisol concentrations between 250 and 450 would only receive stress instructions followed by definitive dynamic testing later [3]. Application of a cut-off value of 250 nmol in our study would have spared 61% of patients from receiving a replacement dose of hydrocortisone at the cost of a reduced sensitivity of 57%. In this case a potential life-threatening adrenal crisis could occur, for which close patient monitoring would be indicated. Based on the low sensitivity in our study we do not advocate the implementation of such a strategy. However, the implementation of multiple early morning (6–9 AM) cortisol measurements would increase sensitivity.

While our study shows that we can reduce unnecessary hydrocortisone prescription in a significant proportion of patients, even with the new cut-off value of 357 nmol/L, 72% of patients would still receive medication unnecessarily for a restricted time period, in our current protocol for 3 months. This raises the questions whether it is justifiable to treat a great number patients to protect the few who develop clinically relevant adrenal insufficiency. Some centers opt for lower cut-off values combined with providing clear stress instructions on when to take medication. Using such an approach, fewer patients will take hydrocortisone on a daily basis, but the patients will be exposed to a small, but greater risk of symptomatic adrenal insufficiency compared to patients following our treatment regimen. We, however, believe that the potential risk of missing adrenal insufficiency by far outweighs the possible risks and burden that are associated with temporary hydrocortisone replacement. New studies evaluating the incidence of symptomatic adrenal insufficiency in patients at risk for postoperative adrenal insufficiency who are not routinely taking hydrocortisone that also include patient reported outcomes on general well-being are urgently needed.

In our center, patients receive definitive testing after a median time of 14 weeks (range 5–57 weeks). In our study timing of metyrapone testing was not significantly related to the outcome of the test. Currently, limited data exist on the optimal timing of definitive testing. English et al. concluded that confirmation testing should be performed not earlier than 6 weeks after surgery as corticotroph function can change over a period of time. On the other hand, one could argue that a postoperative hydrocortisone replacement protocol with a duration of 3 months could induce suppression of the HPA axis at the time of metyrapone confirmation testing, even after 24 h cessation of hydrocortisone. In clinical practice, as is also exemplified in our cohort, the vast majority of patients have normal secretory reserve as reflected by the results of the metyrapone test (76% of patients had normal HPA axis function). This is also in agreement with published reports from other cohorts that have performed confirmatory testing after HC glucocorticoid replacement (20–37.5 mg daily) for 1–6 months and longer after surgery [6, 12, 18–20]. Studies investigating the optimal timing of definitive testing could further reduce the need for unnecessary hydrocortisone replacement. Of note, these results may differ depending on the confirmation test used.

In our study a single early morning postoperative cortisol measurement was the best predictor of adrenal insufficiency. No other clinical factors could be established to further strengthen the prediction for adrenal insufficiency although re-operation could be a risk factor for which a higher power is necessary.

In conclusion, this retrospective study has underlined the importance of reviewing local cut-off values. Our postoperative basal cortisol cut-off value can be lowered to 357 nmol/L, which would result in a 28% reduction in unnecessary hydrocortisone prescription. Even with this cut-off value, 72% of patients would still receive medication unnecessarily. The implementation of a lower cortisol cut-off should first be subject to further prospective evaluation. Further research should aim to establish additional risk factors for adrenal insufficiency and optimal timing of definitive testing to improve the postoperative evaluation of adrenal function.

## Data Availability

The datasets generated during and/or analysed during the current study are available from the corresponding author on reasonable request.
